# Vancomycin treatment and butyrate supplementation modulate gut microbe composition and severity of neointimal hyperplasia after arterial injury

**DOI:** 10.14814/phy2.12627

**Published:** 2015-12-10

**Authors:** Karen J. Ho, Liqun Xiong, Nathaniel J. Hubert, Anuradha Nadimpalli, Kelly Wun, Eugene B. Chang, Melina R. Kibbe

**Affiliations:** ^1^ Department of SurgeryNorthwestern UniversityChicagoIllinois; ^2^ Department of MedicineUniversity of ChicagoKnapp Center for Biomedical DiscoveryChicagoIllinois

**Keywords:** Gut microbiome, neointimal hyperplasia, short chain fatty acids, sodium butyrate

## Abstract

Gut microbial metabolites are increasingly recognized as determinants of health and disease. However, whether host**–**microbe crosstalk influences peripheral arteries is not understood. Neointimal hyperplasia, a proliferative and inflammatory response to arterial injury, frequently limits the long‐term benefits of cardiovascular interventions such as angioplasty, stenting, and bypass surgery. Our goal is to assess the effect of butyrate, one of the principal short chain fatty acids produced by microbial fermentation of dietary fiber, on neointimal hyperplasia development after angioplasty. Treatment of male Lewis Inbred rats with oral vancomycin for 4 weeks changed the composition of gut microbes as assessed by 16S rRNA‐based taxonomic profiling and decreased the concentration of circulating butyrate by 69%. In addition, rats treated with oral vancomycin had exacerbated neointimal hyperplasia development after carotid angioplasty. Oral supplementation of butyrate reversed these changes. Butyrate also inhibited vascular smooth muscle cell proliferation, migration, and cell cycle progression in a dose‐dependent manner in vitro. Our results suggest for the first time that gut microbial composition is associated with the severity of arterial remodeling after injury, potentially through an inhibitory effect of butyrate on VSMC.

## Introduction

Cardiovascular disease from atherosclerosis resulting in acute coronary syndromes, stroke, and critical limb ischemia is a leading cause of morbidity and mortality in the United States (Rosamond et al. 2008), and the economic burden for revascularization procedures continues to rise (Mahoney et al. 2008; Roger et al. 2012). Neointimal hyperplasia is the most common cause of mid‐term restenosis, or recurrent vessel narrowing, after balloon angioplasty/stenting (Weintraub 2007) and bypass graft surgery (Collins et al. 2012). Whereas advances have been made in genetic determinants (Conte et al. 2013), high‐potency statin drugs (Abbruzzese et al. 2004), drug‐coated balloons (Loh and Waksman 2012) and drug‐eluting stents (Lemos et al. 2004) in predicting or reducing the risk of restenosis after cardiovascular interventions, significant residual risk remains, with rates of restenosis reaching 50% at 1 year depending on the arterial bed treated and method of revascularization (Conte et al. 2006; Schillinger et al. 2007).

Neointimal hyperplasia is a form of arterial wound healing. Following arterial injury represented by angioplasty, stenting, or surgery, platelet activation at the site of endothelial denudation leads to leukocyte chemotaxis and infiltration. Vascular smooth muscle cells (VSMC) are then activated to secrete proinflammatory cytokines. Leukocytes, in turn, elaborate cytokines and growth factors that potentiate VSMC proliferation and migration in a paracrine fashion, which ultimately leads to neointimal hyperplasia, a lesion comprising cells (predominantly VSMC and myofibroblasts) and extracellular matrix (Newby and Zaltsman 2000).

A growing body of evidence indicates that gut microbes influence the physiology of their mammalian hosts by signaling through metabolic byproducts such as short chain fatty acids (SCFA). SCFA are produced by the fermentation of dietary fiber by colonic bacteria. The principal SCFA are butyrate, propionate, and acetate (Cummings et al. 1987). Butyrate produced in the colonic lumen is partially metabolized by colonocytes; further uptake occurs in the liver (Cummings et al. 1987). Sodium butyrate has previously been shown to have anti‐proliferative and anti‐migratory effects on VSMC (Ranganna and Yatsu 1997; Ranganna et al. 2000, 2003; Milton et al. 2012; Cantoni et al. 2013). However, while alteration in microbial communities has been associated with the etiopathology of multiple diseases including diabetes and obesity (Turnbaugh et al. 2006; Cani et al. 2008) and gut microbial‐derived metabolites have been shown to promote atherogenesis (Wang et al. 2011), a mechanistic link between gut microbial composition, microbial‐derived SCFA, and neointimal hyperplasia after arterial injury has not previously been made.

Our goal was to establish proof‐of‐concept of a novel link between gut microbes and arterial remodeling after injury. We hypothesized that targeted perturbation of gut microbial composition by antibiotic treatment would modulate circulating levels of butyrate and subsequent susceptibility to neointimal hyperplasia in a rat model of arterial injury. We also sought to demonstrate the effects of sodium butyrate on the proliferation and migration of VSMC in vitro.

## Materials and Methods

### Experimental rats and husbandry

8‐week old male Lewis Inbred rats obtained from Envigo (Indianapolis, IN) were housed in a barrier facility at Northwestern University under a 12‐h light cycle. Standard irradiated rat chow and autoclaved drinking water were provided ad libitum. All animal experiments were approved and conducted in accordance with the Northwestern University Animal Care and Use Committee and the Guide for the Care and Use of Laboratory Animals published by the National Institutes of Health.

### Study design

In order to equilibrate animal housing conditions, bedding (including stool pellets) from all cages were intermixed three times per week for 2 weeks. Rats were then treated for 4 weeks with vancomycin (0.5 mg/mL; Sigma‐Aldrich, St. Louis, MO) and either sodium butyrate (100 mmol/L; Fisher Scientific, Chicago, IL) or sodium‐matched control drinking water. Vancomycin (Lam et al. 2012) and sodium butyrate concentrations (Smith et al. 2013) were based on published work by others. Drinking water solutions were prepared and changed twice per week. Water was provided to animals ad libitum. Animals were weighed on arrival and weekly thereafter. After 4 weeks of oral treatment, all animals underwent left carotid artery balloon angioplasty; the right carotid artery served as the uninjured control artery. Two weeks after angioplasty, all animals were sacrificed for tissue and blood collection. Stool samples were collected upon arrival to the animal facility, prior to the start of water treatment, prior to carotid angioplasty, and on the day of sacrifice. Stool pellets were frozen and stored at −80°C within 1 h of recovery. Whole blood was collected by cardiac puncture prior to perfusion fixation at the time of sacrifice. Serum was prepared by transferring whole blood to serum separator tubes (Life Technologies, Grand Island, NY) for 30 min, followed by centrifugation at 1500 g for 10 min. The supernatant was drawn off and stored at −80°C.

### Rat carotid artery balloon angioplasty

Rats were anesthetized with inhaled isoflurane (0.5–2.5%). Atropine (0.1 mg/kg) was administered subcutaneously to decrease airway secretions. The left neck was shaved and prepped with betadine and 75% alcohol. Following a left paramedian neck incision and dissection of the left common, internal and external carotid arteries, a 2 French Fogarty catheter (Edwards Lifesciences, Irvine, CA) was used for balloon angioplasty injury of the distal common carotid artery as described (Pearce et al. 2008). After removal of the balloon, ligation of the external carotid artery, and restoration of flow to the common and internal carotid arteries, the neck incision was closed in two layers. Carotid arteries were harvested 2 weeks after injury for morphometric analysis.

### Tissue processing

In situ perfusion fixation with phosphate‐buffered saline and 2% paraformaldehyde was performed prior to harvest of both carotid arteries. Arteries were fixed in 2% paraformaldehyde at 4°C for 1 h followed by cryoprotection in 30% sucrose at 4°C overnight, followed by snap freezing in OCT (VWR, Radnor, PA). Five‐*μ*m sections were cut from the distal common carotid artery immediately proximal to the carotid bifurcation.

### Morphometric analysis

Carotid arteries were examined for neointimal hyperplasia after hematoxylin‐eosin staining of artery sections. Digital images of stained sections were obtained using a light microscope with 10× and 40× objectives. Five evenly‐spaced sections from the injured segment were analyzed. Intima and media area were measured using ImageJ software (NIH, Bethesda, MD) after calibration with a ruler.

### Microbial DNA preparation

Stool pellets were collected and stored at −80°C before processing. Microbial DNA was extracted with the PowerSoil DNA Isolation Kit (Mo Bio Laboratories, Carlsbad, CA) following the manufacturer's protocol after 10 minutes of bead‐beating using a Geno/Grinder instrument (Zymo Research, Irvine, CA).

### 16S rRNA‐based analysis of gut microbial composition and diversity

Illumina MiSeq sequencing was performed on community 16S rRNA genes using primers that amplify the V4/V5 hypervariable regions (Earth Microbiome Project 2011). Sequencing was performed at the High‐Throughput Genome Analysis Core (Institute for Genomics & Systems Biology) at Argonne National Laboratory. Assembly and taxonomic assignment was performed on 16S sequence data using the Quantitative Insight Into Microbial Ecology (QIIME) software package against the May 2013 version GreenGenes database at 97% similarity for operational taxonomic units (McDonald et al. 2012). PyNAST was used to align representative sequences (Caporaso et al. 2010a), RDP Classifier to assign taxonomy, and FastTree to determine phylogenetic relatedness (Price et al. 2010). Beta‐diversity was assessed using UniFrac distances, differences were statistically tested using Analysis of Similarity (ANOSIM), and Bon Ferroni‐corrected G‐tests were used to determine which operational taxonomic units significantly discriminate between experimental treatments (Caporaso et al. 2010b).

### Serum butyrate quantification

Butyrate was measured in the serum using a Varian Saturn 2000 GC‐MS‐MS using a published protocol (Renom et al. 2001) with slight modifications. In brief, to 250 *μ*L of serum, an equal volume of nuclease free water was added and acidified with 100 *μ*L of 50% H_2_SO_4_. To the acidified samples, 5 *μ*L of 3 mmol/L isobutyric acid was added as an internal standard. Butyrate was extracted by adding 500 *μ*L of diethyl ether, vortexing for 30 sec, and quickly centrifuging at 5000 g. Five hundred *μ*L of the upper ether layer was transferred to a fresh eppendorf tube and the extraction repeated two more times. One milliliter of the extract was transferred to a glass vial and derivatized with 250 *μ*L of N‐tert‐Butyldimethylsilyl‐N‐methyltrifluoroacetamide (MTBS‐TFA). Samples were run on GC‐MS‐MS within 24 h.

### Cell culture

Aortic VSMC were isolated and cultured from male rats using the collagenase and elastase method as previously described (Yu et al. 1990; Varu et al. 2010). VSMC were maintained and used between passages 4–9. Cells were maintained in DMEM media (Life Technologies) containing 10% FBS (Life Technologies), 100 U/mL penicillin (Life Technologies), 100 *μ*g/mL streptomycin (Life Technologies), and 4 mmol/L L‐glutamine (VWR). When appropriate, cells were quiesced in media containing 1% FBS for 24 h. Cells were incubated at 37°C in 95% air and 5% CO_2_.

### Cell proliferation assay

Aortic VSMC were plated in 24‐well plates (20,000 cells per well) and allowed to adhere for 24 h. Cells were quiesced for 24 h and then treated with 0.5–5 mmol/L sodium butyrate or vehicle in 1% serum media or 10% serum media. 1 mol/L sodium butyrate stock was prepared, sterile filtered, and used within 30 min. All experimental groups were performed in quadruplicate. Cells were harvested for cell counting after 48–72–48 h of treatment. Cells were counted using the MUSE Cell Analyzer Count and Viability Assay Kit (EMD Millipore, Billerica, MA) according to the manufacturer's instructions. All experiments were repeated three times.

### Transwell chemotaxis assay

Aortic VSMC were plated in 10‐cm dishes and allowed to reach confluence. Cells were quiesced for 24 h, lightly trypsinized, incubated with sodium butyrate (0.5–5 mmol/L) or vehicle for 30 min, and then seeded into gelatin‐coated transwells (VWR; 8 μmol/L pores) at a density of 50,000 cells/well. 10% serum media was used as the chemoattractant in the bottom wells. In treatment groups, butyrate was also added to bottom wells. All groups were done in triplicate. After 9–16 h, unmigrated cells were wiped from the top of the membranes. Membranes were fixed in methanol and stained with 4′,6‐diamidino‐2‐phenylindole (DAPI; Life Technologies). The central portion of each membrane was photographed at 20× power with a fluorescence microscope. Three high‐power fields were photographed per membrane. Nuclei were counted and chemotaxis was expressed as a ratio relative to the migrated cells in the negative control (vehicle) group. All experiments were repeated three times.

### Scratch assay

Aortic VSMC were plated in 6‐well dishes (200,000 cells per well) and allowed to adhere for 24 h. Cells were quiesced for 24 h. A scratch was performed with the tip of a pipette. Loosely adherent cells at the edge of the scratch were gently rinsed off. The scratch was photographed at 5× power with a phase contrast microscope. Cells were treated with 0.5–5 mmol/L sodium butyrate or vehicle in 10% serum media for 24 h. Wells were photographed at the same orientation at 24 h. All groups were done in triplicate. All experiments were repeated three times.

### Cell cycle progression assay

Aortic VSMC were plated in 6‐well plates (500,000 cells per well) and allowed to adhere for 24 h. Cells were quiesced for 24 h and then treated with 0.5 mmol/L or 5 mmol/L sodium butyrate or vehicle in 1% serum media or 10% serum media for 24 h. Cells were trypsinized and cell cycle analysis was performed using the MUSE Cell Cycle Assay Kit according to the manufacturer's instructions.

### Endotoxin quantification

Serum was diluted 100‐fold before endotoxin quantification using a chromogenic endotoxin quantification kit (Pierce Biotechnology, Rockford, IL) according to the manufacturer's instructions.

### Statistical analysis

Results are expressed as mean ± SEM. Differences between multiple groups were analyzed using one‐way ANOVA with the Student‐Newman‐Keuls post hoc test for all pairwise comparisons (SigmaStat; SPSS, Chicago, IL). Statistical significance was assumed when *P < *0.05.

## Results

### Oral vancomycin treatment was associated with changes in gut microbial composition and diversity

**Figure 1:**
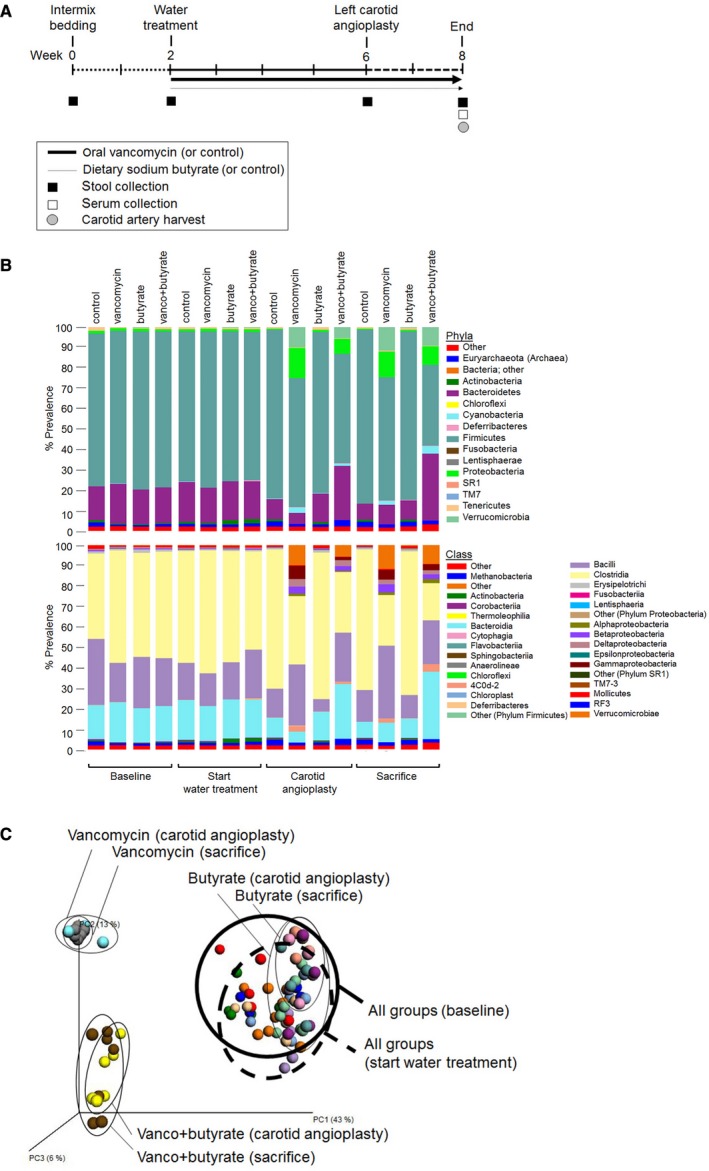
Experimental design and influence of oral vancomycin treatment and dietary butyrate supplementation on gut microbial composition. (A). Study experimental schema. (B). Phylum and class level classification of 16S rRNA gene sequences. Each bar represents the mean relative abundances for each treatment group and timepoint. *N* = 5–8 animals per treatment group. (C). Weighted principal components analysis of the weighted UniFrac distances of stool samples from each rat. Each colored circle corresponds to data from an individual rat at a single timepoint. There were 5–7 rats in each treatment group.

We used oral vancomycin as a means to alter the composition of gut microbiota since it has minimal systemic absorption (Rao et al. 2011) and has defined effects on gut microbial composition (Ubeda et al. 2010; Cho and Blaser 2012) and gut microbial metabolism of SCFA (Yap et al. 2008). Male Lewis Inbred rats underwent a bedding intermixing protocol for 2 weeks to equilibrate housing conditions (Ivanov et al. 2008) before receiving oral vancomycin with either sodium butyrate‐supplemented or sodium‐matched drinking water for 4 weeks (Fig. 1A). Microbial populations present in the stool were monitored by 16S rRNA gene‐based surveys. Phylum‐level and class‐level relative abundances in treatment groups were initially similar, but differences between treatment groups were observed beginning 4 weeks after initiation of vancomycin and vancomycin+butyrate water treatment (Fig. 1B), which persisted until sacrifice. Phylum‐level changes include a decrease in relative abundance of Firmicutes, increase in Bacteroidetes/Firmicutes ratio, and increase in relative abundance of Proteobacteria after vancomycin treatment.

Microbial diversity between treatment groups was evaluated using principal component analysis of the weighted UniFrac distances, which revealed that baseline and prewater treatment communities clustered together (Fig. 1C). In postwater treatment stool, samples separated by the type of treatment, with discrete clusters observed for control, vancomycin, and vancomycin+butyrate treatment. This clustering pattern remained stable for 2 weeks between angioplasty and sacrifice. As the microbial community in the butyrate treatment group clustered with the control group, it was not included in further analyses.

### Oral vancomycin treatment lowered serum butyrate levels and had no long‐term systemic adverse side effects

**Figure 2:**
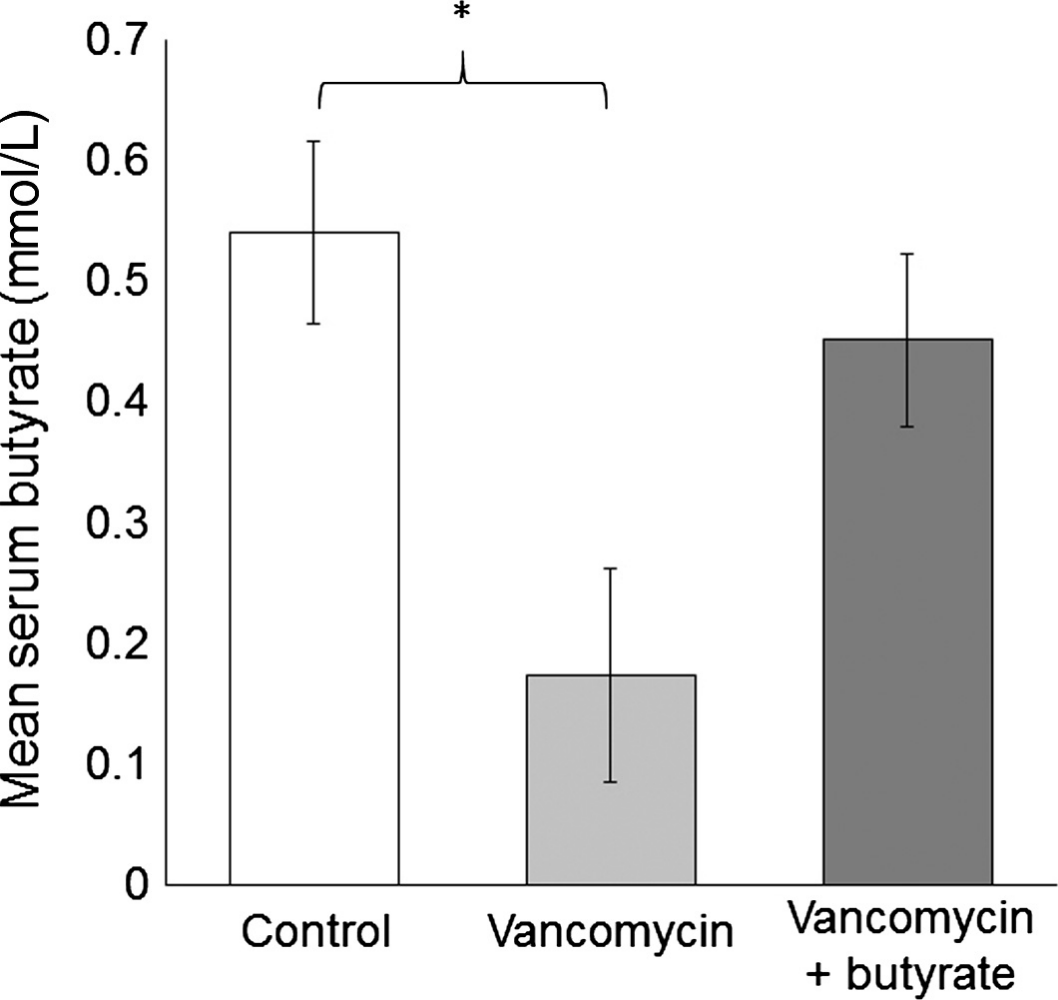
Serum butyrate concentration in each treatment group, expressed as mean ± SEM. Serum was collected by cardiac puncture at the time of sacrifice. *P < 0.05 relative to control group.

**Figure 3:**
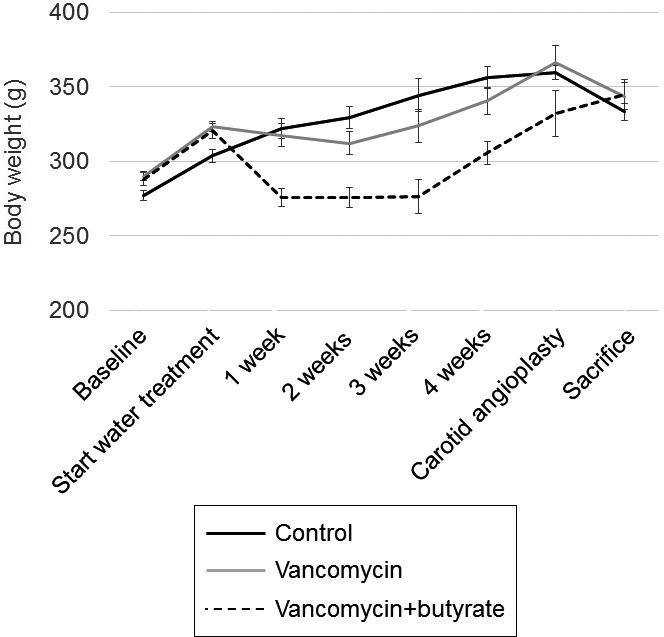
Body weight in each treatment group over time, expressed as mean ± SEM. There were 5–7 rats in each treatment group.

To assess the quantity of butyrate available to peripheral arteries and monitor the effect of treatment on circulating butyrate, we assessed systemic serum butyrate concentration. Serum butyrate concentration at the time of sacrifice in the vancomycin group was 69% lower than the control group (0.17 ± 0.09 mmol/L vs. 0.54 ± 0.07 mmol/L; *P* = 0.02), but was restored to the control level by concomitant supplementation with butyrate (0.45 ± 0.07 mmol/L vs. control; *P* = 0.4) (Fig. 2). To assess systemic effects of vancomycin±butyrate treatment, body weights were obtained throughout the experiment and complete cell count, comprehensive metabolic panel, cholesterol, and triglyceride levels were assessed at the time of sacrifice. As shown in Figure 3, rats treated with vancomycin exhibited weight loss in the first 2 weeks after initiation of treatment. Weight loss was even greater during this early time period after initiation of vancomycin+butyrate treatment. In both these groups, however, weight gain occurred consistently in the subsequent 3 weeks, such that after 5 weeks of treatment, rats in all groups had gained weight compared to baseline and weighed >300 g at the time of carotid angioplasty. Blood tests are shown in Table 1. While there were statistically significant differences among the three treatment groups, none were clinically significant.

**Table 1:** Serum laboratory values (mean ± SEM) in each treatment group

Reference value	Reference value (Envigo, 2007)	Units	Control	Vancomycin	Vancomycin+butyrate	*P* value
White blood cell count	6.0–11.6	×1000	3.6 ± 0.2	3.1 ± 0.2	3.1 ± 0.2	0.23
Hemoglobin	15.6–17.0	g/dL	14.4 ± 0.1	13.9 ± 0.2	13.8 ± 0.1	0.2
Platelet count	683.1–942.7	×1000	702.3 ± 76.0	500 ± 52.7	486.2 ± 51.7	0.09
SGPT	56–41.3	U/L	45.4 ± 4.8	39 ± 3.9	42.4 ± 3.3	0.37
SGOT	45.7–80.8	U/L	123.2 ± 38.0	176.9 ± 68.7	104.4 ± 21.9	0.71
Alkaline phosphatase	151.9–186.9	U/L	191 ± 17.8	200.1 ± 7.9	219.4 ± 6.5	0.08
Total bilirubin	0.3–0.5	mg/dL	0.1 ± 0.0	0.1 ± 0.0	0.1 ± 0.0	0.8
Albumin	3.5–3.9	g/dL	2.9 ± 0.1	2.8 ± .04	2.8 ± 0.02	0.24
Sodium	141.9–152.3	mEq/L	142.8 ± 0.7	145.4 ± 0.5	145.1 ± 0.7	<0.01^a^
Potassium	5.2–6.2	mEq/L	5.5 ± 0.3	4.3 ± 0.1	4.2 ± 0.1	<0.01^a^
Chloride	89.7–100.1	mEq/L	102.6 ± 0.6	102.5 ± 0.3	102.8 ± 0.4	0.75
Blood urea nitrogen	14.1–16.7	mg/dL	18.8 ± 0.4	17.6 ± 0.4	18.7 ± 0.3	<0.01^a^
Creatinine	0.4–0.6	mg/dL	0.3 ± 0.0	0.25 ± 0.02	0.2 ± 0.02	0.04^a^
Glucose	73.4–95.2	g/dL	181.6 ± 13.9	157.3 ± 7.2	161 ± 5.5	0.02^a^
Calcium	10.5–11.1	mg/dL	10.2 ± 0.1	10.1 ± 0.1	10.1 ± 0.1	0.02^a^
Phosphorous	9.9–12.7	mg/dL	5.8 ± 0.2	6.6 ± 0.1	5.4 ± .1	<0.01^a^
Total cholesterol	57.4–74.2	mg/dL	92.4 ± 2.7	88.8 ± 1.9	87.4 ± 2.9	0.03^a^
Triglyceride	72.9–106.9	mg/dL	94 ± 20.4	70.3 ± 7.3	96.4 ± 14.7	0.4

a *P* < 0.05 (ANOVA).

### Oral vancomycin treatment correlated with altered neointimal hyperplasia after arterial injury, which is reversed by butyrate supplementation

**Figure 4:**
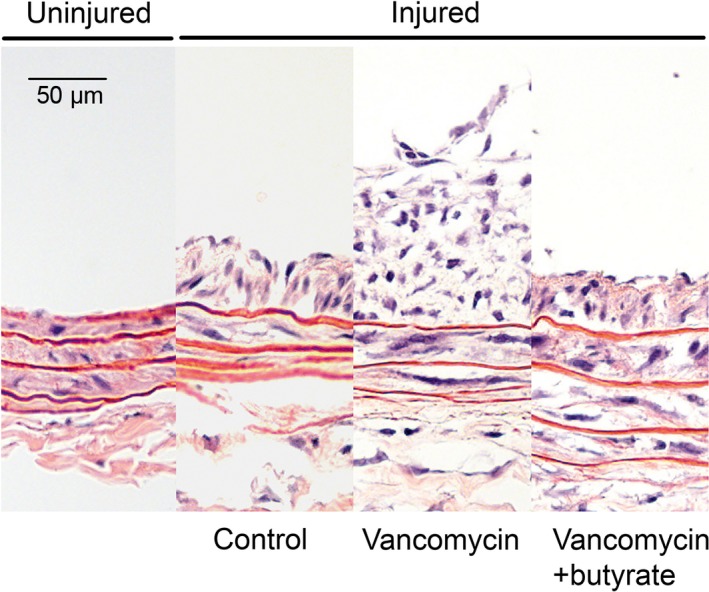
Oral vancomycin treatment exacerbates neointimal hyperplasia development following angioplasty, an effect which is attenuated by concomitant butyrate supplementation. Representative hematoxylin‐eosin‐stained cross sections from an uninjured (right‐sided) carotid artery, injured (left‐sided) carotid artery in control‐treated rat, injured carotid artery in vancomycin‐treated rat, and injured carotid artery in vancomycin+butyrate‐treated rat. Magnification, 50×.

Following balloon angioplasty injury of the carotid artery, neointimal hyperplasia lesions were observed in all three treatment groups at 2 weeks. More neointimal hyperplasia was observed in the vancomycin‐treated rats compared with control rats, which was reversed by concomitant vancomycin+butyrate treatment (Fig. 4; intimal area: 0.032 ± 0.004 mm^2^ control vs. 0.044 ± 0.0043 mm^2^ vancomycin vs. 0.035 ± 0.004 mm^2^ vancomycin+butyrate, *P* = 0.03; intima/media ratio: 0.54 ± 0.06 control vs. 0.62 ± 0.05 vancomycin vs. 0.45 ± 0.06 vancomycin+butyrate, *P* = 0.005). Vancomyin had no effect on uninjured (right‐sided) carotid arteries.

### Serum endotoxin release was low in all treatment groups

Since systemic inflammation induced by endotoxin has been shown to induce neointimal hyperplasia after arterial injury (Danenberg et al. 2002) and since endotoxin is continuously generated in the lumen of the gut by gut bacteria, we measured serum endotoxin at sacrifice to assess the contribution of endotoxin release to differential neointimal hyperplasia formation. Vancomycin treatment did not significantly induce circulating endotoxin concentration (0.034 ± 0.0025 EU/mL) compared to control (0.035 ± .0021 EU/mL control, *P* = 0.85). While the vancomycin+butyrate group had a significantly higher concentration of endotoxin (0.054 ± 0.004 EU/mL, *P* = 0.02 vs. control), this level is far lower than the level used to induce neointimal hyperplasia in vivo (Danenberg et al. 2002; Kong et al. 2013) and to stimulate VSMC in vitro (Ohashi et al. 2000; Lin et al. 2006).

### Sodium butyrate induced a dose‐dependent decrease in rat aortic VSMC proliferation and cell cycle progression

**Figure 5:**
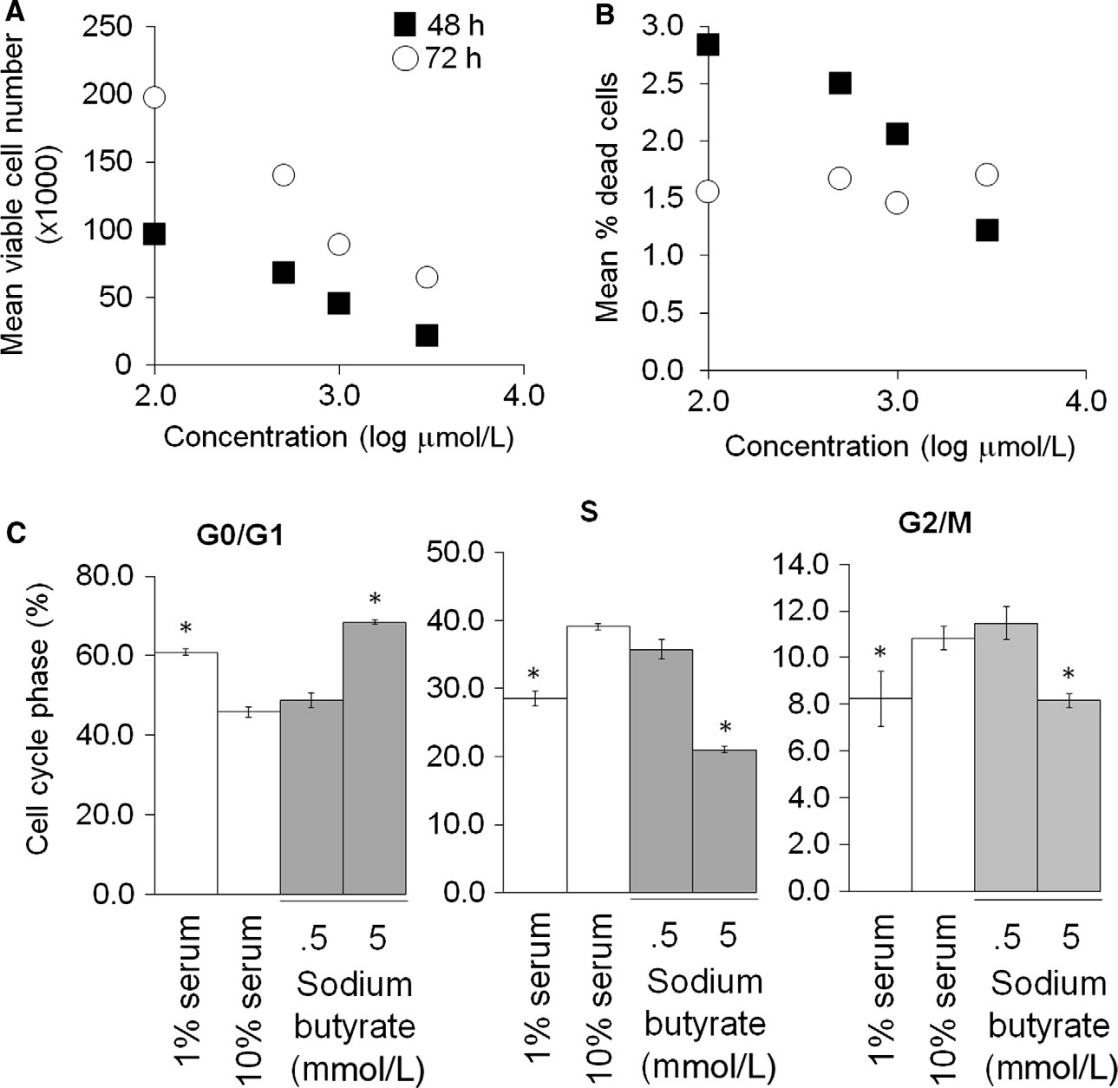
VSMC proliferation, death, and cell cycle progression in presence of sodium butyrate. (A–B). VSMC were treated with sodium butyrate in 10% serum conditions for 48 (black squares) and 72 (white circles) h. Total, viable, and dead cells were counted. Dead cells as a fraction of total cells was calculated. (C). VSMC were treated with sodium butyrate for 24 h and then subjected to cell cycle analysis. Fractions of cells in each cell cycle phase are expressed as mean percentage ± SEM. Representative of *N* = 3. *P < 0.05 relative to 10% serum group.

Aortic VSMC were serum starved for 24 h and subsequently stimulated with 10% serum media and 0.5–5 mmol/L sodium butyrate or vehicle. There was a dose‐dependent decrease in cell proliferation by cell counting after 48 and 72 h of treatment with sodium butyrate (*P* < 0.001 and *P* = 0.02, respectively) (Fig. 5A). After 48 h of treatment, there was a trend toward decreased cell death with increased butyrate dose, but this did not reach statistical significance (*P* = 0.05). At 72 h, there was no difference in cell death across all butyrate doses tested (*P* = 0.95). (Fig. 5B). There was also cell cycle arrest at G_0_/G_1_ in cells treated with 5 mmol/L sodium butyrate for 24 h (Fig. 5C).

### Sodium butyrate induced dose‐dependent decrease in rat aortic vascular smooth muscle cell migration

**Figure 6:**
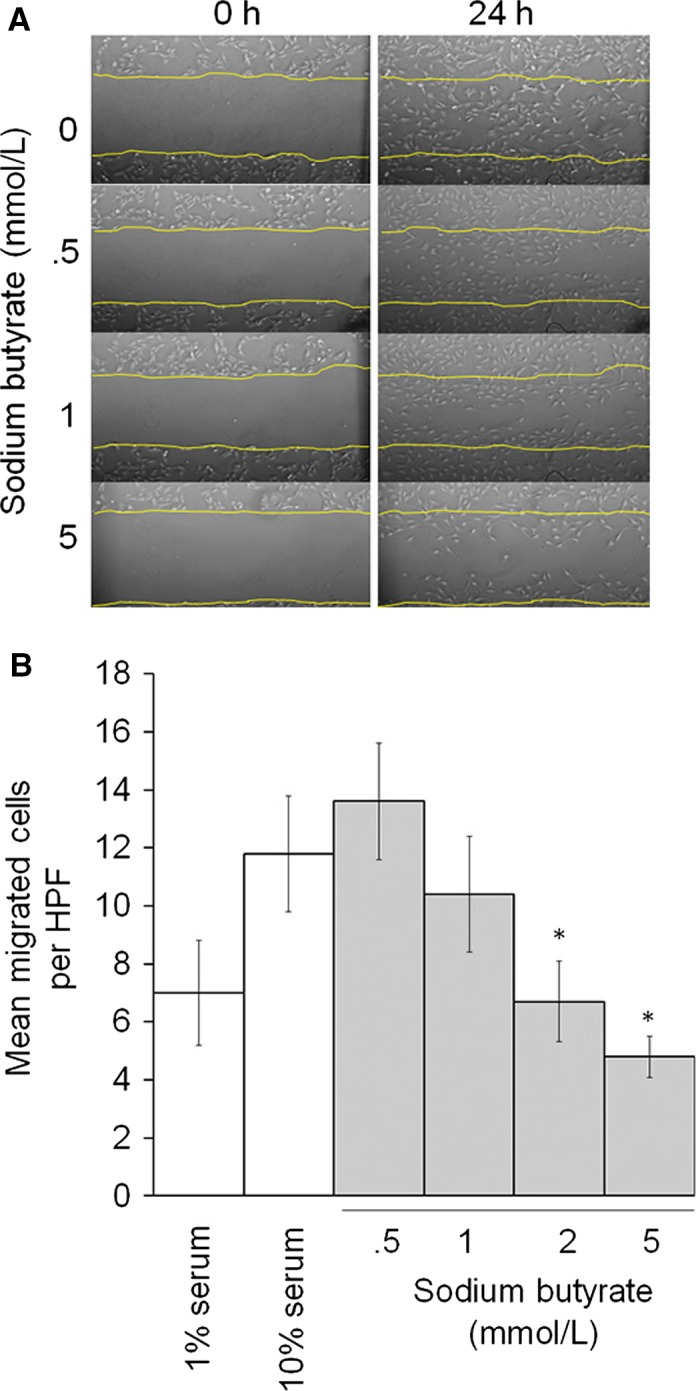
VSMC migration in presence of sodium butyrate. (A). Subconfluent VSMC were treated with sodium butyrate in 10% serum conditions. There is a qualitative dose‐dependent decrease in the number of cells filling in a scratch over 24 h. (B). Migrated VSMC in transwells were counted per high‐powered field (HPF) and migrated cells were expressed as mean number ± SEM. Representative of *N* = 3. *P < 0.05 relative to 10% serum group.

Cell migration was assessed as both migration in a scratch assay and chemotaxis in a transwell assay. As shown in a representative scratch assay (Fig. 6A), there is a dose‐dependent and qualitative decrease in the number of cells repopulating the scratched area after 24 h of treatment with butyrate. Similarly, VSMC treated with butyrate demonstrated less robust chemotaxis to 10% serum in a dose‐dependent manner (Fig. 6B).

## Discussion

Our study correlates shifts in gut microbial composition and serum butyrate concentration with neointimal hyperplasia development after carotid artery balloon angioplasty in rats following treatment with oral vancomycin and dietary supplementation of sodium butyrate. We also observed that sodium butyrate inhibits VSMC proliferation and migration in vitro. Collectively, our data demonstrate proof‐of‐concept that gut microbial composition and butyrate may contribute to the risk for restenosis after cardiovascular interventions.

This is the first report of a potential mechanistic link between the state of host commensal microbes and arterial remodeling after injury. However, others have previously demonstrated clinical and mechanistic links between the cardiovascular system and gut microbes. Examples of these links include the following: Gut microbial metabolism of trimethylamine N‐oxide‐containing dietary nutrients are linked to atherosclerosis and to cardiovascular disease incidence in patients (Wang et al. 2011, 2014; Tang et al. 2013). Vancomycin treatment of rats also correlates with smaller myocardial infarct size, an effect which is also seen after treatment with a probiotic consisting of a leptin‐suppressing *Lactobacillus* strain (Lam et al. 2012). Finally, *Olfr78* null mice, which do not express a receptor for SCFA in the renal juxtaglomerular apparatus, demonstrate hypertension after antibiotic treatment, an effect not observed in *Olfr78* wildtype mice (Pluznick et al. 2013). While systemic inflammation induced by exogenous administration of endotoxin, a product of gram‐negative gut microbes, has been shown to exacerbate neointimal hyperplasia after balloon angioplasty (Danenberg et al. 2002), a direct correlation between alteration in gut microbial composition and neointimal hyperplasia has not previously been reported. Finally, while other investigators have previously demonstrated that butyrate has anti‐proliferative and anti‐migratory effects in VSMC (Ranganna and Yatsu 1997; Ranganna et al. 2000, 2003; Milton et al. 2012; Cantoni et al. 2013), the effect of butyrate on the response to arterial injury in vivo has not been studied.

Antibiotics are a commonly‐utilized method to create global changes in microbial communities in order to study how shifts in microbial composition affects disease (Willing et al. 2011). In our study, we used oral vancomycin treatment as a method to modulate gut microbial composition since it reaches high levels in the intestinal lumen but is poorly absorbed (Khan and Hall 1966; Matzke et al. 1986; Rybak 2006; Cohen et al. 2010; Pfizer 2010; Rao et al. 2011). As such, we surmised that it would affect the composition and diversity of gut microbes without systemic effects, and should thus have an indirect effect on the remodeling peripheral arteries. Vancomycin also induces defined changes in microbe‐derived metabolites in the urine and stool, including increased SCFA concentrations in the stool (Yap et al. 2008). We observed low but not undetectable levels of butyrate in the serum after vancomycin, suggesting that residual butyrate originated from microbes that were not eradicated by vancomycin or from the diet. Notably, we observed no effect of vancomycin on uninjured carotid arteries, suggesting that there is no hyperplastic effect of vancomycin independent of arterial injury. Furthermore, there have been no reports of vancomycin treatment causing neointimal hyperplasia in humans since vancomcyin was approved by the Food and Drug Administration in 1958 (Rybak 2006). As expected from Gram‐positive microbial targeting by vancomycin, we observed a relative decrease in the predominantly Gram‐positive Firmicutes phylum and increase in the Gram‐negative Bacteroidetes phylum after vancomycin treatment. Thus, the significant decrease in serum butyrate concentration after vancomycin treatment that we observed is consistent with reports that well‐described butyrate‐producing bacteria are Gram‐positive Firmicutes (Louis and Flint 2009; Louis et al. 2010). We also observed a relative increase in the Proteobacteria phylum, which encompasses a wide variety of gram‐negative bacteria. The significance of this particular change is not clear, and additional work needs to be done to increase the resolution of this change to higher taxonomic levels. Interestingly, the group that was treated with vancomycin alone did not have a significant increase in serum endotoxin level compared to the control group despite the relative increase in the Proteobacteria phylum. The group that was treated with vancomycin+butyrate, however, had a nearly 60% increase in serum endotoxin level compared to the control group. While this finding could suggest an increase in systemic inflammation, we observed a similar level of neointimal hyperplasia development in this group compared to control and the level of endotoxin we measured is more than one order of magnitude less than what was used to induce neointimal hyperplasia in endotoxemia (Danenberg et al. 2002).

Butyrate has been shown previously to have anti‐proliferative and anti‐migratory effects on VSMC (Ranganna et al. 1995, 2000, 2003; Ranganna and Yatsu 1997; Cantoni et al. 2013). Ranganna et al. (1995) observed that 3–8 mmol/L butyrate inhibits serum‐induced proliferation of aortic VSMC without alteration of cell viability (Ranganna et al. 2000) in PDGF‐dependent manner (Ranganna et al. 1995) while Cantoni et al. (2013) demonstrated that 5–50 mmol/L butyrate induced significant inhibition of PDGF‐induced proliferation, migration, and cell cycle progression of pulmonary artery VSMC. We tested butyrate in vitro at a lower dose range of 0.5–5 mmol/L, which is on the order of serum concentrations observed in rats and humans, depending on diet and diurnal fluctuations (Demigne and Remesy 1985; Wolever et al. 1997). These data, together with in vivo correlations between elevated serum buyrate and attenuated neointimal hyperplasia, suggest that butyrate may inhibit neointimal hyperplasia by directly inhibiting VSMC.

We did not observe any long‐term systemic toxicity associated with vancomycin or butyrate treatment. We observed gradual but transient weight loss in the group treated with vancomycin+butyrate, which we attribute to a period of accommodation necessary for the rats to become accustomed to the odor and taste of butyrate (Canani et al. 2011; Mattace Raso et al. 2013; Pituch et al. 2013). We addressed this by changing the water frequently. We also did not observe any diarrhea (loose or watery stool pellets) in any of the 151 stool pellets that were collected over all timepoints in all treatment groups.

We were uncertain about the effect of enteral butyrate supplementation on microbial composition and serum butyrate concentration. However, as we were primarily interested in the effect of gut microbial shifts on serum butyrate and neointimal hyperplasia, and enteral butyrate treatment alone did not significantly alter microbial composition from control drinking water, we did not include this group in the analysis.

Our study has several limitations. Our data are principally physiologic observations, but will hopefully be fertile ground for future studies into mechanistic links between microbes, butyrate, and neointimal hyperplasia development to determine if the findings are correlative or causative or viable therapeutic targets Furthermore, we have no data on the functional output of the microbial communities stratified by treatment group beyond serum butyrate levels, which also limits our evaluation of how the treatment groups affected other microbe‐dependent metabolites. Antibiotic treatment is a blunt but useful tool (Kostic et al. 2013) for altering the composition and diversity of gut microbes, and although these data do not reveal a genera or strain of microbes that predispose or protect against neointimal hyperplasia, this will be the objective of future work. Finally, although we focused on butyrate, it is possible that the other major SCFA, such as acetate and propionate, also affect neointimal hyperplasia development.

In summary, our analyses correlate features of microbiome dysbiosis and susceptibility to neointimal hyperplasia after arterial injury. Opportunities for translational medical research and investigations into the mechanism of the effect of butyrate on the host and VSMC in particular warrant future studies that incorporate 16S rRNA gene surveys to recognize the full potential of the gut microbiome and ultimately guide therapeutic strategies for manipulating the microbiome to prevent and treat cardiovascular disease.

## Conflict of Interest

None declared.
