# Corrigendum

**DOI:** 10.14814/phy2.13035

**Published:** 2016-11-14

In Ho et al. (2015), the author's name, Nathaniel J. Hubert, was incorrect and should have read, Nathaniel A. Hubert.

We apologise for the typographical error.
